# Temporal Requirement for the Protective Effect of Dietary Cholesterol against Alcohol-Induced Vasoconstriction

**Published:** 2020-10-20

**Authors:** Olga Seleverstov, Kelsey North, Maria Simakova, Shivantika Bisen, Alexandra Bickenbach, Zoran Bursac, Alex M. Dopico, Anna N. Bukiya

**Affiliations:** 1 Department of Pharmacology, Addiction Science, and Toxicology, College of Medicine, The University of Tennessee Health Science Center, Memphis, Tennessee, United States of America; 2 Department of Biostatistics, Stempel College, Florida International University, Miami, Florida, United States of America

**Keywords:** High-cholesterol diet, Vascular smooth muscle, Binge drinking, Pressurized cerebral artery

## Abstract

Moderate-to-heavy episodic alcohol drinking resulting in 30-80 mM of ethanol in blood constricts cerebral arteries and constitutes a risk factor for cerebrovascular disease. Alcohol-induced constriction of cerebral arteries *in vivo* and *ex vivo* has been shown to be blunted by dietary cholesterol (CLR) in a rat model of a high-CLR diet. Such protection has been proposed to arise from the high-CLR diet-driven increase in blood CLR levels and accompanying buildup of CLR within the cerebral artery smooth muscle. Here we used a rat model of high-CLR feeding *in vivo* and pressurized cerebral arteries *ex vivo* to examine whether the degree and time-course of alcohol-induced constriction are related to blood CLR levels. We demonstrate that subjecting young (3 weeks-old, 50 g) male Sprague-Dawley rats to a high- CLR feeding up to 41 weeks, resulted in an age-dependent increase in total blood CLR levels, when compared to those of age-matched rats on isocaloric (control) chow. This increase was paralleled by a high-CLR diet-driven elevation of blood low-density lipoproteins whereas high-density lipoprotein levels matched those of age-matched, chow-fed controls. Alcohol-induced constriction was only blunted by high-CLR dietary intake when high-CLR chow was taken for up to 8-12 and 18-23 weeks. However, alcohol-constriciton was not blunted when high-CLR chow intake lasted for longer times, such as 28-32 and 38-41 weeks. Thus, alcohol-induced constriction of rat middle cerebral arteries did not critically depend on the total blood CLR levels. Alcohol-induced constriction seemed unrelated to the natural, progressive elevation of the total blood CLR level in control- or high-CLR-fed animals over time. Thus, neither the exogenously nor endogenously driven increases in blood CLR could predict cerebral artery susceptibility to alcohol-induced constriction. However, we identified a temporal requirement for the protective effect of dietary CLR against alcohol, that could be governed by the young age of the high- CLR chow recipients (3 weeks of age) and/or the short duration of high-CLR chow feeding lasting for up to 23 weeks.

## 1. Introduction

Episodic alcohol consumption with blood alcohol levels (BAL) reaching 30-80 mM is a well- recognized risk factor for cerebrovascular disease and increased incidence of stroke [1,2]. At these BALs, alcohol constricts cerebral arteries in several experimental species, including humans [1,3]. Alcohol-induced constriction does not require neuronal network activity, bioactive factors from the systemic circulation, or a functional endothelium, as the alcohol effect is observed in isolated, de-endothelialized and *ex vivo*-pressurized cerebral arteries [4].

Pathological consequences of brain ischemia include neurological and sensory deficits, motor incoordination, dizziness and sleepiness [5,6]. These symptoms closely mimic those associated with heavy alcohol intoxication [7]. Yet factors that determine or regulate alcohol effect on cerebral artery diameter remain understudied.

Cholesterol (CLR) is a characteristic constituent of Western diets [8]. Increased dietary intake of CLR and an eventual increase in blood CLR levels pose a significant risk for numerous systems and organs, including cerebral arteries [9,10]. Despite the deleterious effect of a high-CLR diet on vascular function, we have shown that dietary CLR protects against alcohol-induced constriction of rat cerebral arteries *in vivo* [11]. Moreover, this protection is fully replicated *ex vivo* using pressurized arteries dissected from rats on a high-CLR diet and compared to that observed with *ex vivo* artery data from animal donors on a control diet (standard rodent chow) [11]. Such protection has been proposed to arise from the high-CLR diet-driven increase in blood CLR levels and the accompanying buildup of CLR within cerebral artery smooth muscle [11]. Indeed, a high-CLR diet resulted in CLR accumulation within cerebral artery smooth muscle itself [11]. An increase in cerebral artery smooth muscle CLR can also be achieved by *in vitro* CLR enrichment of de-endothelialized cerebral arteries from rats on standard rodent chow [12,13].

This *in vitro* CLR enrichment results in a diminished constriction of rat cerebral arteries by 50 mM alcohol (ethanol, EtOH) [14]. Moreover, the protective effect of CLR is removed upon *in vitro* depletion of excessive CLR from cerebral arteries of rats that received high-CLR chow for 18-22 weeks [11]. These studies revealed that the blood CLR level could be used a potential biomarker for cerebral artery’s susceptibility to alcohol-induced constriction. However, whether there is a dependence of cerebral artery constriction by alcohol on time-course and/or levels of blood CLR has never been formally tested. Using a wide range of blood CLR levels, the present study answers such question by unveiling that the protective effect of dietary CLR against alcohol is restricted to younger animals and is only observed when high-CLR feeding does not exceed certain duration.

## 2. Materials and Methods

### 2.2 Ethical Aspects of Research

Care of animals and experimental protocols were reviewed and approved by the Institutional Animal Care and Use Committee of the University of Tennessee Health Science Center, which is an institution accredited by the Association for Assessment and Accreditation of Laboratory Animal Care International (AAALACi).

### 2.3 *In vivo* procedures and animal euthanasia

**Figure 1:**
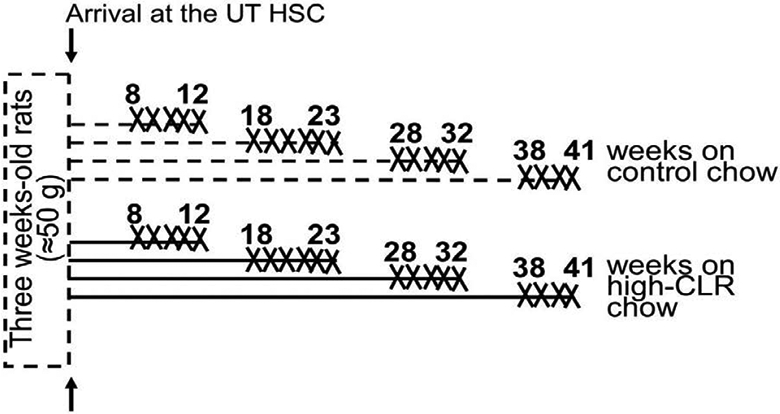
Timeline of in vivo dietary intervention and *ex vivo* tissue harvesting. Timelines of feeding with control chow are dashed, high-CLR feeding is in solid lines. “X” represents number of weeks during which animals in each group were progressively sacrificed for the purpose of blood collections and tissue harvesting. UT HSC: University of Tennessee Health Science Center.

Three weeks-old male Sprague-Dawley rats (average weight 50 g) were purchased from Envigo (former Harlan) company (Indianapolis, IN, USA). Upon arrival to the University of Tennessee Health Science Center, animals were acclimated for 3 days and then randomly divided into eight age-matched groups. Four of these groups were placed on a standard rodent chow (Teklad 22/5, Teklad Global Diets® by Envigo, Indianapolis, IN, USA), while the remaining four groups received high-CLR chow diet (2% CLR, Teklad Global Diets® by Envigo, Indianapolis, IN, USA) *ad libitum* for up to 12, 23, 32, or 41 weeks (Figure 1). Animal euthanasia for the purpose of blood and tissue collection began several weeks prior to reaching the experimental endpoint in each group.

Thus, animals in the first group were progressively sacrificed (please see “X” marks in Figure 1) during weeks 8-12 of receiving control chow. In the second group, animals were progressively sacrificed during weeks 18-23 of feeding. In the third group, animal sacrifice took place during weeks 28-32, while in the fourth group animals were sacrificed during weeks 38-41. Similar timelines were applied to the four groups of animals receiving high-CLR chow. Animals were deeply anesthetized with isoflurane via inhalation in a jar, then decapitated using a guillotine. Blood was collected for determination of total blood CLR, low-density lipoprotein (LDL), and high-density lipoprotein (HDL) levels. The brain was removed from the skull and placed into an ice-cold phosphate buffered saline for consequent dissection of cerebral arteries.

### 2.4 Cerebral artery diameter measurements *ex vivo*

On the day of the experiment, rats were euthanized as described above. Middle cerebral arteries (MCAs) were dissected out on ice under a microscope (Nikon SMZ645 by Nikon^®^, Melville, NY, USA) and cut into segments. A segment was cannulated at each end in a temperature-controlled, custom-made perfusion chamber.

The chamber with the cannulated artery was continuously perfused by a Dynamax RP-1 peristaltic pump (Rainin Instr., Oakland, CA, USA), at a rate of 3.75 mL/min with physiologic sodium saline (PSS) of the following composition (mM): 119 NaCl, 4.7 KCl, 1.2 KH_2_PO_4_, 1.6 CaCl_2_, 1.2 MgSO_4_, 0.023 EDTA, 11 glucose, 24 NaHCO_3_. PSS was equilibrated at pH 7.4 with a 21/5/74% mixture of O_2_/CO_2_/N_2_ and maintained at 35-37°C. Arteries were monitored with a video camera (Sanyo VCB-3512T by Sanyo, Osaka, Japan) attached to an inverted microscope (Nikon Eclipse TS100, Nikon^®^, Melville, NY, USA). The artery wall external diameter was measured using the automatic edge-detection function of IonWizard software (IonOptics Inc., Waltham, MA, USA), with traces being digitized at 1 Hz. Steady- state changes in intravascular pressure were achieved by elevating an attached reservoir filled with PSS and were monitored using a pressure transducer (Living Systems Instrumentation, St Albans City, VT, USA). Arteries were first incubated at an intravascular pressure of 10 mmHg for 15 min. Then, intravascular pressure was increased to 60 mmHg and held steady to evoke myogenic tone development and maintenance throughout the experiment. Artery viability was validated by a robust vasoconstrictive response to a high-KCl solution of the following composition (mM): 63.7 NaCl, 60 KCl, 1.2 KH_2_PO_4_, 1.2 MgSO_4_, 0.023 EDTA, 11 glucose, 24 NaHCO_3_, 1.1 CaCl_2_, equilibrated at pH 7.4 with a 21/5/74% mixture of O_2_/CO_2_/N_2_. After high-KCl solution washout, ethyl alcohol (EtOH) was diluted in (30 mL) PSS to 50 mM and applied to the artery via chamber perfusion.

### 2.3 Determination of blood CLR levels

Whole blood was collected from the animal trunk, placed into a polypropylene centrifuge tube, and left for 10 min on ice. Tubes were centrifuged at 4°C, 2400 rpm for 15 min. Clear supernatant was collected and stored at −80°C. Blood lipid levels were determined using a Cobas Mira biochemistry analyzer (Roche, Basel, Switzerland) at the University of Tennessee HSC Endocrinology laboratory on a fee-for-service basis.

### 2.4 Chemicals

Alcohol (ethyl alcohol, 100% purity, 200 proof) was purchased from American Bioanalytical Inc. (Natick, MA, USA). CLR was purchased from Avanti Polar Lipids (Alabaster, AL, USA). All other chemicals were purchased from Sigma-Aldrich (St. Louis, MO, USA). Alcohol was freshly diluted in PSS immediately before application to the artery. Each pressurized artery segment was exposed to alcohol only once to avoid reduced responsiveness to multiple applications of alcohol.

### 2.5 Data analysis

Artery diameter data were analyzed using IonWizard 4.4 software (IonOptics Inc., Waltham, MA, USA). Baseline diameter was obtained by averaging diameter values during 3-5 minutes of recording immediately before drug application and following 10-15 min of washout. A drug-induced change in arterial diameter was determined from the peak effect obtained during drug application.

Further analysis, plotting, and fitting of data were conducted using Origin 2020 (OriginLab Corp., Northampton, MA, USA) and InStat 3.0 (GraphPad Software Inc., San Diego, CA, USA). When the number of observations in each dataset exceeded 10, Gaussian distribution of the data was tested using a Kolmogorov-Smirnov test. For normally distributed variables, a comparison of means between groups was conducted using a two-sample t-test. In all other cases (number of observations <10, or unsatisfactory result from Kolmogorov-Smirnov test), the distribution of data was presumed to be non-Gaussian. Thus, nonparametric statistical tests such as Mann-Whitney U test and Kruskal-Wallis test with Dunn’s multiplicity adjustment were used to compare the distributional equality according to the experimental design (also see individual figure captions for details of statistical analysis for each dataset). Associations between continuous variables were tested with simple linear regression models and Pearson correlation coefficient. Unless stated otherwise, comparison of two datasets was performed using a two-tailed analysis. Results were considered significant at an alpha level of 0.05. Throughout text, data are expressed as the mean ± standard error (SE). In the figures, error bars represent standard deviation (SD). In all bar graphs, individual data-points represent blood samples or MCA segments; each blood sample or MCA segment was obtained from a separate animal.

## 3. Results

### 3.1 High-CLR chow increased total blood CLR level through an increase in LDL

**Figure 2:**
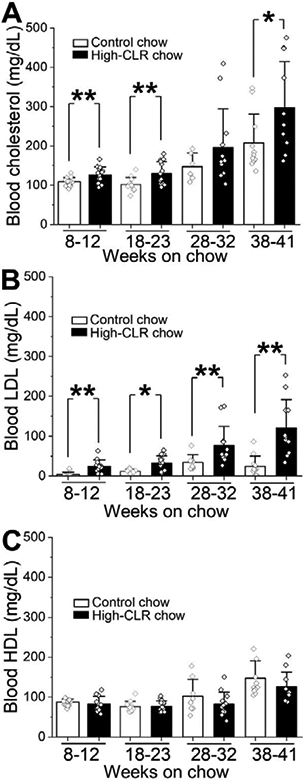
High-CLR chow increased blood total CLR level via an increase in LDL. Here and in all other bar graphs, data are presented as mean±SD. In all bar graphs, individual data-points represent blood samples or middle cerebral artery (MCA) segments, each blood sample or MCA segment was obtained from a separate animal. For this figure, blood samples were obtained from the following number of rats: 8-12 weeks on control chow (n=15), 8-12 weeks on high-CLR chow (n=14), 18-23 weeks on control chow (n=12), 18-23 weeks on high-CLR chow (n=15), 28-32 weeks on control chow (n=8), 28-32 weeks on high-CLR chow (n=12), 38-41 weeks on control chow (n=11), 38-41 weeks on high-CLR chow (n=11). A. Data showing changes in blood total CLR by the high-CLR chow feeding of increasing duration. P=0.009 by two-sample t-test (8-12 weeks on chow); p=0.009 by two-sample t-test (18-23 weeks on chow); p=0.034 by two-tail Mann-Whitney U test (38-41 weeks on chow). B. Data showing changes in blood low-density lipoprotein (LDL) by the high-CLR chow feeding of increasing duration. P=0.0001 by two-tail Mann-Whitney U test (8-12 weeks); p=0.01 by two-tail Mann-Whitney U test (18-23 weeks); p=0.003 by two-tail Mann-Whitney U test (28-32 weeks), p=0.0003 by two-tail Mann- Whitney U test (38-41 weeks). C. Data showing changes in blood high-density lipoprotein (HDL) by the high-CLR chow feeding of increasing duration. There were no significant changes detected between control- and high-CLR chow-fed groups.

Rat feeding with a high-CLR chow *ad libitum* resulted in a statistically significant increase in total blood CLR levels over those of age-matched rats on control chow (Figure 2A). Notably, after 28-32 weeks of a high-CLR diet, blood CLR level exceeded 190 mg/dL, thus meeting the definition of a borderline pathologic CLR level in humans [14]. After 38-41 weeks on the high-CLR diet, blood CLR levels exceeded 240 mg/dL, thus constituting clinically defined hypercholesterolemia in humans [15]. At any given time-interval (range: 8-41 wks.), feeding rats with high-CLR chow resulted in blood LDL levels that were significantly increased when compared to those from rats on control chow (Figure 2B). However, blood HDL values did not differ significantly between control and high-CLR chow-fed groups after any duration of feeding (8-41 weeks) (Figure 2C).

### 3.2 Alcohol-induced constriction was only blunted by high-CLR dietary intake at early time-points

Analysis of MCA constriction by 50 mM EtOH was performed using *ex vivo* pressurized MCAs from rats that were offered control versus high-CLR chow *ad libitum* for increasing periods of time (8-41 weeks). Rats on high-CLR chow grew to a larger size compared to their littermates receiving control food. The weight gain became apparent after 18-23 weeks on high-CLR chow. In this group, animals weighed on average 452.3±8.3 g (n=6) versus 418.9±7.8 g weight of their age-matched littermates on control chow (n=5) (p=0.0173 by 2-tail Mann-Whitney test). Such difference in weight may potentially result in larger cerebral arteries in the animals on high-CLR chow. However, our earlier work established that there was no correlation between artery external diameter and degree of alcohol-induced constriction in rats weighting between 50 and 440 g [16].

**Figure 3:**
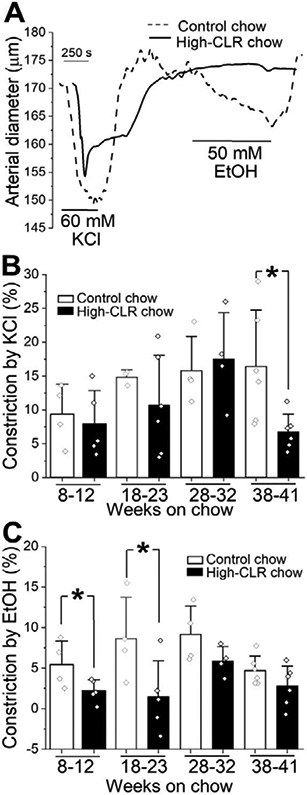
Alcohol-induced constriction was only blunted by high-CLR dietary intake at early time- points. A. Original traces of MCA diameter showing blunting of alcohol-induced constriction in artery segment from animal subjected to a high-CLR diet for 23 weeks when compared to animal donor on control chaw. EtOH: ethyl alcohol (ethanol). Arterial general contractility was probed with 60 mM KCl. B. Data comparing constriction by 60 mM KCl between control- and high-CLR chow-fed groups with increasing duration of feeding. Number of data-points is as follows: 8-12 weeks on control chow (n=4), 8- 12 weeks on high-CLR chow (n=5), 18-23 weeks on control chow (n=3), 18-23 weeks on high-CLR chow (n=6), 28-32 weeks on control chow (n=4), 28-32 weeks on high-CLR chow (n=4), 38-41 weeks on control chow (n=6), 38-41 weeks on high-CLR chow (n=6). *Statistically significant difference from control. P=0.009 by two-tail Mann-Whitney U test. C. Data comparing constriction by 50 mM EtOH between control- and high-CLR chow-fed groups with increasing duration of feeding. Number of data- points is as follows: 8-12 weeks on control chow (n=4), 8-12 weeks on high-CLR chow (n=5), 18-23 weeks on control chow (n=4), 18-23 weeks on high-CLR chow (n=5), 28-32 weeks on control chow (n=4), 28-32 weeks on high-CLR chow (n=4), 38-41 weeks on control chow (n=6), 38-41 weeks on high-CLR chow (n=6). *Statistically significant difference from control. P=0.03 by one-tail Mann-Whitney U test (8-12 weeks); p=0.03 by one-tail Mann-Whitney test (18-23 weeks).

Following established protocols, MCAs were pressurized at 60 mmHg for development and maintenance of myogenic tone. At the beginning of each experiment, artery segment contractility was verified by the artery contractile response to 60 mM KCl (Figure 3A). High-CLR chow did not modify KCl responses of MCA with the exception of feeding for 38-41 weeks (Figure 3B). At this duration of high-CLR feeding, KCl-induced constriction dropped from 16.4±3.4% in control (n=6) to 6.8±1.1% of pre-KCl pressurized artery diameter in the high-CLR fed group (n=6) (p=0.0087, 2-tailed Mann-Whitney test, (Figure 3B).

The high-CLR diet significantly increased total blood CLR levels in groups that were subjected to high-CLR feeding for 8-12, 18-23, and 38-41 weeks (Figure 2A). Remarkably, EtOH-induced constriction was significantly blunted only in groups subjected to high-CLR feeding for 8-12 and 18-23 weeks (Figure 3C).

### 3.3 Alcohol-induced constriction of rat MCAs did not parallel changes in total blood CLR levels

**Figure 4:**
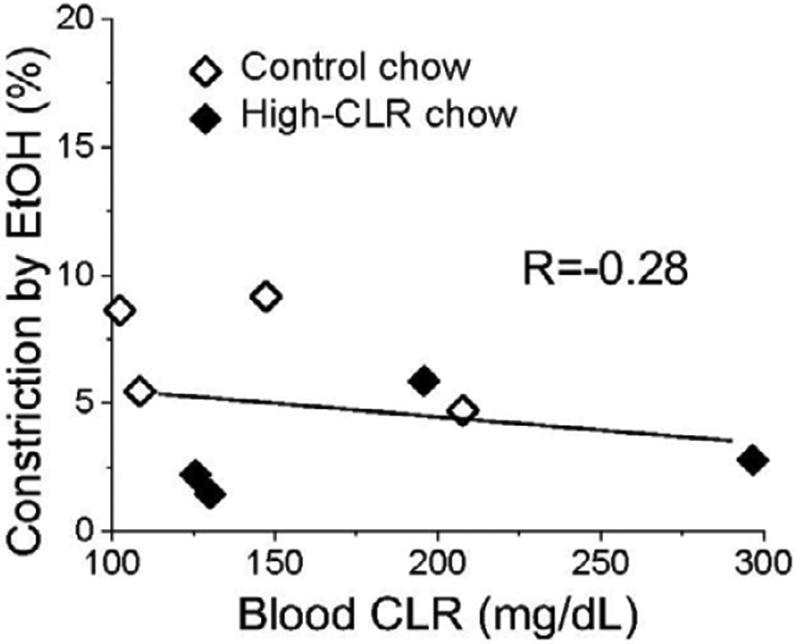
EtOH-induced constriction of rat MCAs did not correlate with blood total CLR level. Plot represents averaged data for each control- and high-CLR chow-fed group of animals with corresponding averaged blood total CLR levels at 8-12, 18-23, 28-32, and 38-41 weeks of control (hollow symbols) and high-CLR chow feeding (black symbols). Data were fitted with simple linear function to determine Pearson’s (correlation) coefficient R.

Overall, there was no correlation between the degree of EtOH-induced constriction and total blood CLR levels when control and high-CLR groups were evaluated together using linear regression (R= −0.28, Figure 4). Attempts to utilize a non-linear regression analysis were unsuccessful.

### 3.4 Alcohol-induced constriction was unaffected by a progressive and natural elevation of the total blood CLR levels

**Figure 5:**
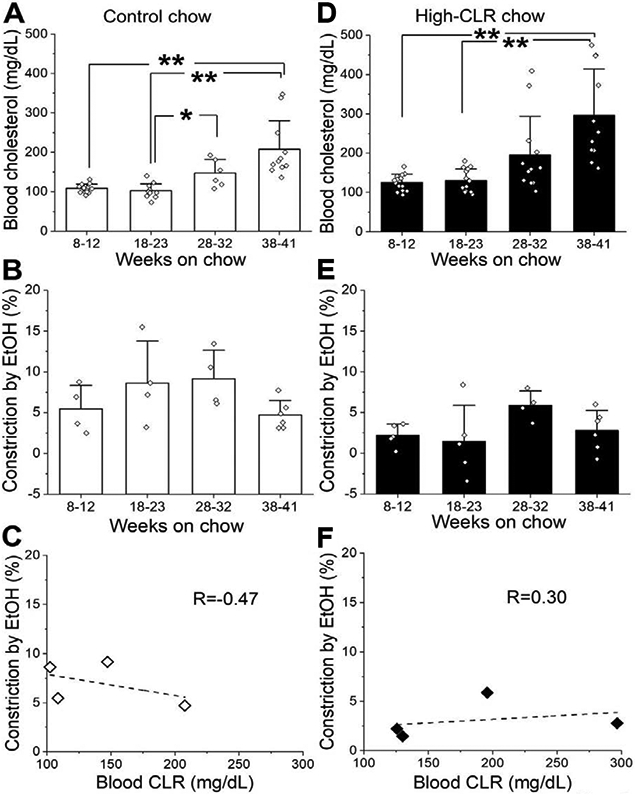
Alcohol-induced constriction was unaffected by progressive natural elevation of blood total CLR level. For number of dataponts in panels A-B and D-E, please refer to legends of Figures. 2 and 3. A. Data showing progressive increase in blood total CLR in rats fed by control chow. *P<0.05 and **P<0.01 by Kruskal-Wallis test with Dunn’s post-test adjustment for multiplicity B. Data depicting constriction by 50 mM EtOH in control chow-fed rats. There were no statistically significant changes detected. C. Plot representing averaged data for each control chow-fed group of animals at any given duration of feeding and corresponding averaged blood total CLR level. Data were fitted with simple linear function to determine Pearson’s (correlation) coefficient R. D. Data showing progressive increase in blood total CLR in rats fed high-CLR chow. **P<0.01 by Kruskal-Wallis test with Dunn’s post-test adjustment for multiplicity. E. Data depicting constriction by 50 mM EtOH in high-CLR chow-fed rats. There were no statistically significant changes detected. F. Plot representing averaged data for each high-CLR chow-fed group of rats and corresponding averaged blood total CLR level. Data were fitted with simple linear function to determine Pearson’s (correlation) coefficient R.

We noticed that there was a significant progressive increase in the total blood CLR levels over time, in rats offered control chow (Figure 5A). For instance, total blood CLR levels in rats fed control chow for 31-41 weeks almost doubled the values from 8-12 weeks on control chow: 207.7±22.0 mg/dL (n= 11) vs. 108.5±2.8 mg/dL (n=15), respectively (p<0.001 by Kruskal-Wallis test with Dunn’s post-test, Figure 5A). Despite a natural increase in total blood CLR levels as rats aged, EtOH-induced constriction did not differ across the control groups (Figure 5B). Thus, there was no temporal parallelism of EtOH-induced constriction and total blood CLR levels in the control groups. The dissimilar time-course of the two variables under consideration is underscored by their lack of correlation (R=−0.47, Figure 5C).

A similar progressive increase in blood CLR was observed in high-CLR chow-fed rats (Figure 5D). Blood CLR levels in these animals after 38-41 weeks of feeding reached 296.6±35.5 mg/dL (n=11), which is significantly higher than 125.7±5.6 mg/dL after to 8-12 weeks of feeding (n=14) (p<0.001 by Kruskal-Wallis test with Dunn’s post-test) or 130.1±7.9 mg/dL after 18-23 weeks of feeding (n=15) (p<0.001 by Kruskal-Wallis test with Dunn’s post-test). Despite a progressive increase in total blood CLR levels as rats grew and consumed high-CLR chow, EtOH-induced constriction did not differ across the high-CLR chow-fed groups (Figure 5E). Thus, there was no temporal parallelism between EtOH-induced constriction and total blood CLR levels in the high-CLR chow groups. The dissimilar time-course of the two variables under consideration is underscored by their lack of correlation (R=0.30, Figure 5F).

It is noteworthy that the increase in total blood CLR levels in the control group at 28-32 weeks of feeding when compared to the control group at 18-23 weeks of feeding (Figure 5A), led to the total blood CLR level observed in the high-CLR groups at 8-12 and 18-23 weeks (Figure 5D). Yet, EtOH-induced constriction was nearly identical in the two control groups (18-23 versus 28-32 weeks of feeding, Figure 5B), while high-CLR feeding in both age (younger) groups, resulted in diminished constriction by 50 mM EtOH (Figure 3C).

## 4. Discussion

Using a rat model, in the current work we have established that blood CLR levels do not readily predict susceptibility of MCA constriction in response to alcohol. Moreover, we report for the first time that the protection of dietary CLR against alcohol-induced vasoconstriction is only effective at a specific age and duration of high-CLR feeding.

In the course of the study, we utilized a high-CLR diet to trigger a CLR increase in rat blood. Dietary CLR intake triggers modifications in blood lipid profile in humans and represents an approach that is widely used for manipulation with blood CLR in laboratory animal models [17-20]. Our findings were obtained using a rat model of high-CLR diet. Compared to humans, rats have lower level of cholesteryl ester transfer protein, which is thought to be responsible for cholesterol clearance from HDL particles [20,21]. Thus, rats have a higher HDL pool in the total blood CLR, but a lower LDL count than humans [20,22]. Whether low levels of LDL in rats constitutes a limitation of the rat model remains to be established. However, dietary-driven hypercholesterolemia in rats is widely used for studies of CLR metabolism [23,24]. Moreover, the rat model of high-CLR dietary intake has been proven useful for prior studies of CLR-alcohol interactions in the cerebrovascular system [11,25]. In the present study, rats were offered a high-CLR chow starting from a young age (3 weeks-old).

The longer feeding of animals with the high-CLR chow gradually increased blood CLR levels, and covered a wide range of blood CLR levels starting from 100 mg/dL and reaching up to 300 mg/dL (Figure 2A). It is noteworthy that 100 mg/dL corresponds to a blood CLR level within the targeted range for patients with a high risk for cardiovascular disease [26,27]. While we did not study blood CLR levels below 100 mg/dL, such low CLR levels are rarely seen in healthy individuals [28]. On the other hand, blood CLR levels exceeding 240 mg/dL are reflective of hypercholesterolemia in humans [26,27].

In our experimental model, the increase in blood CLR was associated with a drastic rise in blood LDL levels, in response to high-CLR chow, when compared to control feeding in all groups (Figure 2B). The responsiveness of blood CLR levels to high-CLR dietary content makes high-CLR feeding a valuable tool for studying the consequences of elevated CLR on physiology and pathology [28].

In the present work, we utilized rat model of high-CLR dietary intake to evaluate blood CLR levels and alcohol-induced constriction at corresponding blood CLR levels/time-points in a search for a potential biomarker for cerebral artery susceptibility to alcohol-induced constriction. The search for an easily screened marker constitutes a topic of high clinical importance. Indeed, alcohol levels resulting from excessive alcohol consumption lead to cerebral artery constriction in adults of several species, including humans [1,3,29], and are associated with higher incidences of stroke and stroke-related deaths [30-32]. Our focus on MCA over other cerebral vessels was dictated by the fact that MCA irrigates the largest territory of the brain and accounts for the largest share of brain perfusion when compared to other cerebral arteries stemming from the circle of Willis [33]. MCA’s irrigation territory covers subcortical nuclei and lateral parts of brain hemispheres [34]. Considering major role played by MCA in brain irrigation, it is not surprising that perturbations in MCA tone and diameter are at the center of cerebrovascular disorders, including ischemia and strokes [35-37]. Consistent with epidemiological studies and previous reports on cerebral arteries in many species, we detected on average a 5-10% decrease in MCA diameter by 50 mM alcohol across our control groups or rats (Figure 5B) [1,3,4]. This alcohol level is detected in human blood after moderate-to-heavy alcohol intake [7]. Noteworthy, seemingly small reduction in cerebral artery diameter is expected to result in a massive drop in brain perfusion, as changes in artery diameter are related to changes in cerebral blood flow by a factor of ~3-4 [38].

Consistent with our earlier observations [11], high-CLR chow diminished alcohol-induced constriction of cerebral arteries after 18-23 weeks of feeding (Figure 3A and 3C). In this study, however, we also observed the protective effect of high-CLR feeding against alcohol-induced vasoconstriction at a shorter duration of high-CLR feeding (8-12 weeks, Figure 3C). This protection seems rather specific, as the contractile response of a cerebral artery to a non-selective vasoconstrictor (60 mM KCl) remained unchanged by high-CLR chow in both groups (8-12 weeks, 18-23 weeks, Figure 3B).

Surprisingly, a further increase in the duration of high-CLR feeding to 28-32 and 38-41 weeks did not produce protection against alcohol, even though the high-CLR chow triggered a sharp increase in total blood CLR level when compared to age-matched animals fed control chow (Figure 2A and 3C). There are two possible explanations for the observed specificity of the protective effect during a particular time- point of feeding. First, high-CLR chow feeding requires a specific duration (up to 23 weeks) in order to provide effective protection against alcohol. When this duration is exceeded, protection is lost, possibly due to adaptative changes at the organismal or tissue levels. Second, the duration of high-CLR chow feeding does not matter, but rather the key determinant of such specificity is the particular age at which animals are fed with high-CLR chow, making them more resistant to alcohol-induced constriction of cerebral arteries. Conceivably, these two explanations are not mutually exclusive. However, we favor the latter possibility: indeed, in the control chow-fed group, blood CLR levels kept rising in the absence of high-CLR feeding, and this rise was observed later than 23 weeks of feeding (Figure 5A). The progressive increase in CLR levels with growth and age was not a surprise, as it has been widely reported in young or middle-aged humans [39-43]. Yet, in our experiments, the rise in blood CLR did not render protection against alcohol-induced constriction (Figure 5B). Unless endogenously driven elevation of blood CLR by natural aging versus exogenously introduced CLR via high-CLR feeding is based on different molecular mechanisms, it is reasonable to conclude that there is a specific time at which an elevation in blood CLR level is most efficient in rendering protection against alcohol. Perhaps, the fact that blood CLR levels in the 8-12 and 18-23 week groups were relatively low compared to 28-32 and 38- 41 weeks (Figure 2A and 5A) could have played a role in the distinct resistance of these animals. Considering that there is a buildup of CLR within cerebral artery smooth muscle in response to high-CLR feeding [11], there may be a physical saturation of tissue with CLR. Saturation in the ability of cerebral artery smooth muscle to accumulate CLR has been reported by our group in studies with *in vitro* CLR enrichment of rat cerebral arteries using the chemical carrier methyl-beta-cyclodextrin [12]. In this case, elevation in blood CLR beyond a specific level would not result in a proportional increase in the amount of CLR present in the arterial wall. Thus, the impact of a further elevation in blood CLR level on alcohol-induced vasoconstriction would be minimal (if any). Another possibility is that elevation of blood CLR at different time-points of life (young versus middle age) engages different molecular players that modulate the effect of alcohol on cerebral artery diameter. These players may represent, for example, channel-forming and regulatory proteins of calcium- and voltage-gated potassium channels of large conductance (BK type), transient receptor potential vanilloid receptor 1 (TRPV1), or ryanodine receptor (RyR). The aforementioned protein complexes have age-sensitive dynamics [16,44,45] and have been identified by our group as molecular targets of alcohol in cerebral arteries [3,4,46-48]. More specifically, BK channels which represent one of the major ion channel targets of alcohol in vascular smooth muscle, show age-dependent changes in the expression of accessory beta1 subunit [16]. From 3 to 15 weeks of age, BK channel beta1 subunit amount in the plasma membrane of rat cerebral artery myocytes is progressively increased [16]. Yet in older rats (28-30 months of age), BK channel beta1 subunit transcript is decreased when compared to 3-4 months-old animals [49]. Therefore, there seems to be an optimal time-point during natural development and growth, at which BK channel betal subunit level and function reach their maximum. Notably, the BK channel beta1 subunit enables BK channel inhibition by alcohol [3], and also defines sensitivity of vascular smooth muscle BK channel to CLR [50]. In the present study, we did not probe CLR effect on alcohol-induced constriction of cerebral arteries in very young animals. If dietary CLR failed to protect against alcohol-induced constriction in this group, it would mean that the CLR protection against the effect of alcohol on BK currents and cerebral artery diameter requires a maximal amount of the beta1 subunit. Our earlier work, however, seems to refute this interpretation, as CLR antagonism of alcohol effect on cerebral artery diameter was observed in beta1 subunit-lacking (*KCNMB1* knock-out) mice [14]. Thus, temporal requirement for CLR protection against alcohol-induced constriction of cerebral arteries likely involves molecular players additional to BKs. These players could diminish BK channel sensitivity to the CLR- alcohol interaction(s) in older animals or compensate for loss of constriction, if the CLR-alcohol interaction(s) at BK channels remains effective. Future studies will shed light on the precise molecular mechanisms that govern the temporal requirements of the protective effects of dietary CLR against alcohol-induced constriction.

## 5. Study significance

Although natural aging and high-CLR dietary intake were effective in increasing blood total blood CLR levels, the latter did not apparently correlate with susceptibility of cerebral arteries to alcohol-induced constriction. However, we detected a temporal requirement for the protective effects of dietary CLR against alcohol. In perspective, our study may pave the way for the future identification of molecular mechanisms that govern CLR-alcohol interactions in the control of cerebral artery diameter, which will be helpful in combating the devastating consequences of episodic drinking on cerebral circulation.

## Abbreviations

BAL: blood alcohol level
CLR: cholesterol
EtOH: ethanol
HDL: high-density lipoprotein
LDL: low-density lipoprotein
MCA: middle cerebral artery
PSS: physiologic sodium saline
